# Enrollee Characteristics in an Intensive Tobacco Dependence Treatment Program: The Relationship of Race and Sex to Demographic Factors and Tobacco Use Patterns

**DOI:** 10.3389/fpsyt.2019.00112

**Published:** 2019-03-21

**Authors:** Thomas J. Payne, Christine E. Sheffer, Natalie W. Gaughf, Monica J. Sutton, Holly H. Peeples, Okan U. Elci, Jennie Z. Ma, Alan Penman, Karen M. Crews

**Affiliations:** ^1^Department of Otolaryngology and Communicative Sciences, University of Mississippi Medical Center, Jackson, MS, United States; ^2^Department of Health Behavior, Roswell Park Cancer Institute, Buffalo, NY, United States; ^3^Academic Counseling, University of Mississippi Medical Center, Jackson, MS, United States; ^4^Department of Pediatrics, University of Mississippi Medical Center, Jackson, MS, United States; ^5^Department of Family Medicine, University of Mississippi Medical Center, Jackson, MS, United States; ^6^Center for Biostatistics, University of Mississippi Medical Center, Jackson, MS, United States; ^7^Department of Public Health Sciences, University of Virginia, Charlottesville, VA, United States; ^8^Professor Emeritus, University of Mississippi Medical Center, Jackson, MS, United States

**Keywords:** tobacco treatment, racial differences, gender differences, demographic factors, tobacco use

## Abstract

Intensive tobacco treatment programs offer many advantages relative to other treatment options, particularly for more complex patients, e.g., highly nicotine dependent, or those with medical and psychiatric symptoms and disorders. Efforts to better understand those who choose to enroll in these programs, particularly regarding the characteristics they possess known to mediate outcomes, are important considerations in tailoring available services. In this study, we examined how participants differed on key descriptive and tobacco use variables within race (i.e., African-American, Caucasian) and sex subgroups. Baseline characteristics from a large group of consecutive program enrollees were examined across targeted subgroups. Strong racial effects and some sex effects were noted for marital status, education, employment and health insurance status, alcohol consumption, presence of medical and psychiatric disorders, as well as participant tobacco use patterns and tobacco use rates of family, friends and coworkers. The differences in participant tobacco use measures across race and sex factors remained significant after adjusting for the confounding effects of all other covariates. These findings have implications for characterizing key patient subgroups who present at tobacco treatment clinics. Such information may contribute to options for tailoring treatment regimens.

## Introduction

Tobacco use remains the most preventable cause of death and disability in the USA and globally (1, 2). In the long term, substantially reducing the number of new users is essential to ending the tobacco epidemic. In the short-to-medium term, evidence-based treatments have been shown to effectively increase quit rates, reduce morbidity and mortality, and contain healthcare costs, such that tobacco intervention remains the gold standard of cost effectiveness (3–9).

A variety of characteristics have been identified as negative predictors for successfully achieving abstinence. These include a high level of nicotine dependence, presence of co-morbid psychiatric and substance dependence disorders, greater psychological distress, lower socioeconomic status / education level / income, lower levels of social support, and increased alcohol consumption, among others (10–16). Most of this evidence is based on samples of community smokers, or those being seen in outpatient medical services [e.g., (17)]. There have been far fewer efforts to evaluate the characteristics of those enrolling in more intensive tobacco treatment programs. Foulds et al. (18) noted that several factors predicted 26-week outcome in 1,021 tobacco treatment clinic enrollees, including demographics (age, education, number of children, insurance status), as well as tobacco-related factors (time to first use of tobacco, night awakening to smoke, readiness to quit, number of clinic contacts). Of interest was the fact that no sex effect was detected; and while there was a short-term race effect, this was no longer evident in the 6-month multivariate analysis. In an unpublished report of the NJ Quitcenters outcomes for a diverse population clients, no sex effect was noted, but Caucasians were more successful quitting at 6-month follow-up than African-Americans (19). Burke et al. (20) conducted a similar study with a much larger sample (*N* = 6,824), and also noted that expected factors were associated with treatment outcome. However, given the focus of the study, baseline characteristics were incorporated primarily for their value as covariates in treatment outcome analyses. In addition, all participants were enrolled at a single site, limiting study generalizability. Analyses of patient subgroups were limited. Sheffer et al. (21) conducted a treatment outcome predictor study of 2,350 patients enrolled in multiple statewide clinic sites. An in-depth analysis of similar baseline factors as predictors was conducted, and greater detail regarding those factors was provided relative to other research. The sample was primarily white and married, low income, had a partner who smoked, achieved less than high school education, had a lengthy smoking history, was highly nicotine dependent and reported elevated levels of stress. Most were highly motivated to quit tobacco. Sheffer et al. (22), in comparing in-person enrollees to telephone quitline callers, noted similar characteristics across samples. Once again, both of these studies included a primarily Caucasian sample, and conducted minimal subgroup analyses.

In an attempt to improve treatment outcomes, a more in-depth examination of key presenting characteristics is needed. In particular, understanding how such factors differentially characterize key population subgroups should more readily allow for intervention tailoring. As the need for and value of intensive Tobacco Treatment Specialist-based services becomes more widely acknowledged and employed, such data will become increasingly important in our efforts to triage individuals to achieve maximal outcomes and enhance cost effectiveness / benefit.

This study represents an attempt to begin to fill this knowledge gap by conducting an in-depth analysis of key characteristics in a large treatment-seeking sample. Such findings can improve our understanding of those who attend programs, as well as establish a baseline against which future studies may be compared. Our primary goal was to examine the degree to which important descriptive and tobacco use characteristics identified in previous research differed among racial and sex subgroups when examined in our statewide treatment sample.

## Materials and Method

### Participants

All English-speaking tobacco users aged 18 years or older who identified as African-American (AA) or European-American (EA) and enrolled for treatment of tobacco dependence at any of our 21 sites across the state of Mississippi from 2009 to 2012 were included. Participants were excluded from treatment if they were unable to participate in a self-management oriented intervention (e.g., had an unstable, serious psychiatric, or medical condition).

### Tobacco Dependence Treatment

Participants who enrolled in the ACT Center Statewide Network of Tobacco Treatment Clinics were informed that this program provided standardized, evidence-based, multicomponent cognitive-behavioral therapy plus medication for tobacco dependence. The intervention approach is consistent with the tenets of the U.S. Public Health Service Clinical Practice Guideline (5). The treatment is similar to that provided in other statewide programs (19, 21, 22), and consisted of 6 weekly closed group 60–75 min sessions of manual-driven, multicomponent, cognitive-behavioral therapy, followed by approximately six individual follow-up sessions scheduled through 1-year post-treatment. Participants provided an expired breath sample at each visit for measurement of carbon monoxide, and were given the option of using medications. Medication options included nicotine replacement, bupropion, and varenicline, either alone or in combination, typically for a 3–6 month duration. All treatment was provided at no cost to participants, utilizing funds allocated from the state master settlement agreement, and awarded via legislative appropriation and a grant from the Mississippi State Department of Health Office of Tobacco Control. All treatment programs were housed within outpatient services of 18 medical centers or 3 large outpatient clinics. Locations were distributed across the state in urban, suburban, and rural locations.

### Procedures

Tobacco users typically had an initial contact with a clinic site by telephone. Most sought treatment services based on friend or family recommendation, or direct referral by a healthcare provider. All were scheduled for an intake, at which time informed consent for treatment was obtained, followed by administration of assessment measures via computer or on paper per the participants' preference and the resources available at the treatment site. Assistance was provided as needed. Administration time typically ranged from 30 to 45 min. Shortly after the assessment was completed, participants met with a Certified Tobacco Treatment Specialist who discussed the assessment findings, described the treatment program, and scheduled participants for their first treatment session.

### Measures

The components of the ACT Center comprehensive self-report intake assessment addressed in this study included: (a) demographic characteristics, (b) medical and psychiatric history, (c) tobacco use history, (d) Fagerström Test for Nicotine Dependence (FTND; (23)), (e) tobacco use by friends, family, and coworkers, and (f) alcohol consumption (typical number of standard drinks consumed per week).

### Data Analysis

Descriptive analyses were conducted on all measures and are reported as means and standard deviations, or frequencies. Tobacco use measures included menthol use, FTND score, age at smoking initiation, age when starting regular smoking, number of years of regular smoking, average daily smoking rate, maximum daily smoking rate, and age when achieved maximum daily rate. Menthol use was considered as a binary outcome in logistic regression and all other tobacco use measures as continuous outcomes in the linear regression. The primary interest in the regression analyses was to evaluate the effects of race, sex, and their interaction on these outcomes. The analyses were further adjusted for the impact of potentially confounding factors, including age, education, marital status, employment, insurance status, number of smokers in household, spouse/partner use of tobacco, and percent of friends and coworkers using tobacco. All participants enrolled in the study period were included in the univariate analyses and those with missing data were excluded from multivariable analyses (<5%). Results from multivariable regression analyses were expressed as odds ratios for menthol use and mean difference for continuous tobacco use outcomes along with their corresponding 95% confidence intervals [CI]. A *p*-value < 0.05 was considered statistically significant. Stata (version 12.1; Stata Corporation, College Station, TX) was used to conduct all statistical analyses.

## Results

### Sample Characteristics

All demographic and other descriptive variables were significantly different by Race × Sex at *p* < 0.001 except where indicated (Table 1). Participants (*N* = 11,292) were a socio-demographically diverse group of primarily middle-aged men and women. Only 16% rated their health as very good, 4% as excellent, and 79% fair to good. Average BMI was in the overweight range (28.7 [6.8] kg/m^2^). Most (53%) had achieved a high school education, with 19% attaining a college degree. Only 41% were full-time employed, and 8% part-time. Significant differences were also noted across all medical and psychiatric conditions except kidney disease, and more than 30% of the sample reported lung disease, cardiovascular disease, and high blood pressure. Significant rates were also endorsed for allergies (27%), mental health conditions (18%), and diabetes (12%). Importantly, 46% reported feeling depressed for 2 weeks in the past 12 months (screening item for major depressive disorder), 39% reported ongoing depressive symptoms over the past 2 years, 42% had received treatment for depression, 39% had received treatment for anxiety, and 16% had received treatment for other psychiatric conditions. Twenty-nine percent had no health insurance, 29% were on Medicaid and/or Medicare, and 43% reported private insurance.

**Table 1:** Descriptive characteristics.

	**Total *N* = 11,292**	**AA Men *N* = 982 (8.7%)**	**AA Women *N* = 1,661 (14.7%)**	**EA Men *N* = 3,200 (28.3%)**	**EA Women *N* = 5,449 (48.3%)**
**Age [Mean (SD)]**	47.85 (12.7)	48.91 (12.92)	49.78 (11.42)	47.30 (13.31)	47.38 (12.6)
**AGE, CATEGORY**					
18–34	2,078 (18%)	156 (16%)	207 (12%)	670 (21%)	1,045 (19%)
35–64	8,240 (73%)	738 (75%)	1,334 (80%)	2,229 (70%)	3,939 (73%)
65+	935 (08%)	86 (09%)	118 (07%)	285 (09%)	446 (08%)
**EDUCATION**					
Up to 11th grade	1,787 (16%)	264 (27%)	328 (20%)	507 (16%)	688 (13%)
GED	1,378 (12%)	75 (08%)	166 (10%)	402 (13%)	735 (14%)
HSD /Some College	5,909 (53%)	521 (54%)	836 (51%)	1,676 (53%)	2,876 (53%)
College graduate (4 year) or higher	2,117 (19%)	110 (11%)	310 (19%)	586 (18%)	1,111 (21%)
**MARITAL STATUS**					
Single	2,595 (23%)	338 (35%)	689 (42%)	690 (22%)	878 (16%)
Married /Living Together	5,431 (48%)	435 (44%)	431 (26%)	1,846 (58%)	2,719 (50%)
Separated /Divorced /Widowed	3,226 (29%)	206 (21%)	534 (32%)	657 (21%)	1,829 (34%)
**EMPLOYMENT**					
Full-time	4,580 (41%)	313 (32%)	580 (36%)	1,513 (48%)	2,174 (40%)
Part-time	846 (08%)	63 (07%)	135 (08%)	190 (06%)	458 (08%)
Student /Homemaker /Retired /Disabled	4,469 (40%)	462 (48%)	697 (43%)	1,102 (35%)	2,208 (41%)
Unemployed	1,262 (11%)	131 (14%)	218 (13%)	361 (11%)	552 (10%)
**INSURANCE**					
Commercial	3,401 (35%)	189 (23%)	362 (25%)	1,053 (39%)	1,797 (38%)
Medicaid	846 (09%)	104 (13%)	210 (15%)	135 (05%)	397 (08%)
Medicare	1,153 (12%)	112 (14%)	146 (10%)	359 (13%)	536 (11%)
Medicare + Medicaid	764 (08%)	116 (14%)	187 (13%)	162 (06%)	299 (06%)
None	2,779 (29%)	222 (28%)	394 (28%)	784 (29%)	1,379 (29%)
Other	741 (08%)	62 (08%)	131 (09%)	222 (08%)	326 (07%)
**ALCOHOL DRINKING STATUS**					
Current	4,450 (41%)	478 (51%)	665 (42%)	1,347 (44%)	1,960 (37%)
Former	4,255 (39%)	366 (39%)	576 (36%)	1,331 (43%)	1,982 (38%)
Never	2,132 (20%)	94 (10%)	353 (22%)	383 (13%)	1,302 (25%)
**ALCOHOL CONSUMPTION PER WEEK**					
< 1	2,285 (38%)	134 (22%)	343 (38%)	571 (32%)	1,237 (46%)
1–3	1,950 (32%)	206 (33%)	383 (43%)	481 (27%)	880 (32%)
4–7	790 (13%)	129 (21%)	107 (12%)	257 (14%)	297 (11%)
≥8	1,018 (17%)	153 (25%)	66 (07%)	500 (28%)	299 (11%)
**OVERALL HEALTH**					
Excellent	347 (04%)	27 (03%)	30 (02%)	140 (05%)	150 (03%)
Very good	1,598 (16%)	111 (13%)	174 (12%)	474 (17%)	839 (17%)
Good	4,189 (42%)	330 (39%)	551 (39%)	1,194 (42%)	2,114 (44%)
Fair	2,905 (29%)	287 (34%)	542 (38%)	745 (26%)	1,331 (28%)
Poor	857 (09%)	83 (10%)	118 (08%)	265 (09%)	391 (08%)
BMI kg/m^2^ [Mean (SD)]	28.7 (6.79)	28.5 (6.25)	30.73 (7.44)	28.43 (5.96)	28.27 (7.03)
Obesity	1,279 (11%)	46 (05%)	212 (13%)	224 (07%)	797 (15%)
Lung /respiratory disease	3,748 (33%)	223 (23%)	499 (30%)	917 (29%)	2,109 (39%)
Cardiovascular disease^*^	3,543 (31%)	333 (34%)	550 (33%)	1,007 (31%)	1,653 (30%)
Diabetes	1,371 (12%)	163 (17%)	310 (19%)	322 (10%)	576 (11%)
High blood pressure	3,661 (32%)	478 (49%)	853 (51%)	943 (29%)	1,387 (25%)
Cancer	898 (08%)	81 (08%)	137 (08%)	202 (06%)	478 (09%)
Mental health	2,039 (18%)	105 (11%)	226 (14%)	447 (14%)	1,261 (23%)
Alcohol/Substance abuse	791 (07%)	104 (11%)	74 (04%)	304 (10%)	309 (06%)
Kidney disease^**^	336 (03%)	34 (03%)	39 (02%)	94 (03%)	169 (03%)
Allergy	3,060 (27%)	127 (13%)	357 (21%)	690 (22%)	1,886 (35%)

Test for Race by Sex interaction p < 0.001 for all analyses, except

* *p < 0.05*,

** *ns based on Likelihood-ratio test*.

*% calculated based on number of responses (missing data not included)*.

AA participants were older than EA, and about twice as many were single. A smaller percentage of AA men had attained a college degree or higher relative to other groups. AA males were least likely to be full-time employed, and EA males were most likely. There was no difference across groups in the percentage possessing health insurance, but approximately twice as many AA participants were enrolled in Medicaid, while EAs were more likely to have private insurance.

Regarding measures of health, more EA smokers reported their overall health as excellent or good relative to AA smokers, although all values were fairly low. AA women had a higher mean BMI compared with others, and 13% were classified as obese. EA participants indicated higher rates of chronic lung and respiratory disorders, whereas diabetes and hypertension were endorsed by AA participants at nearly twice the rate of EA participants.

A greater percentage of AA participants were current drinkers, however overall reported consumption levels were modest. Twice as many men than women reported a history of drug or alcohol dependence; there were no racial differences. EA women reported a higher frequency of mental health diagnoses (23%) than all other groups (Table 1).

### Participant Tobacco Use

All participant tobacco use measures were significantly different by Race x Sex at *p* < 0.001 (Table 2). Participants were overall highly dependent on tobacco with a mean FTND score of 5.6 (2.3) and an average of 27.3 (13.0) years of regular smoking, starting at age 16.0 (4.7), and becoming regular smokers at 18.1 (5.4) years. The average number of cigarettes smoked per day in the past year was 20.9 (10.6), and 38% of participants reported smoking menthol cigarettes. A large proportion of participants lived with spouses / partners who smoked (40%), 41% indicated working in an environment where 25% or more co-workers smoked, and 77% indicated that 25% or more of their friends smoked (Table 2).

**Table 2:** Participant and others' tobacco use factors.

		**Total *N* = 11,292**	**AA Men *N* = 982 (8.7%)**	**AA Women *N* = 1,661 (14.7%)**	**EA Men *N* = 3,200 (28.3%)**	**EA Women *N* = 5,449 (48.3%)**
**PARTICIPANT TOBACCO USE**	Menthol	4,057 (38%)	766 (83%)	1,281 (81%)	587 (20%)	1,423 (27%)
	FTND	5.6 (02.26)	5.01 (02.19)	5.04 (02.13)	5.93 (02.26)	5.69 (02.26)
	Age first cigarette	15.95 (04.72)	16.16 (04.12)	17.88 (05.15)	14.94 (03.84)	15.88 (04.93)
	Age started smoking regularly	18.09 (05.36)	18.28 (04.38)	20.22 (05.76)	16.91 (04.38)	18.09 (05.68)
	Age reached maximum daily rate	24.76 (09.73)	24.12 (08.34)	26.77 (10.04)	23.54 (08.71)	25.03 (10.29)
	Maximum daily rate	34.65 (16.41)	26.39 (013.7)	22.21 (11.90)	43.62 (17.83)	34.81 (13.84)
	Number years regularly smoking	27.34 (13.03)	27.56 (13.57)	25.89 (12.57)	28.53 (13.89)	27.07 (12.52)
	Average daily rate, past year	20.94 (10.61)	16.96 (09.88)	14.98 (08.52)	25.04 (11.76)	21.08 (09.53)
**OTHERS' TOBACCO USE**	**NUMBER IN HOUSEHOLD**					
	0	1,793 (16%)	195 (21%)	324 (20%)	458 (15%)	816 (15%)
	1	3,236 (29%)	239 (25%)	440 (27%)	960 (31%)	1,597 (30%)
	2-3	4,207 (38%)	352 (37%)	571 (35%)	1,254 (40%)	2,030 (38%)
	4 or more	1,774 (16%)	160 (17%)	274 (17%)	454 (15%)	886 (17%)
	**SPOUSE/PARTNER TOBACCO USE**					
	No	3,909 (36%)	540 (57%)	625 (39%)	1,270 (41%)	1,474 (28%)
	Yes	4,363 (40%)	230 (24%)	498 (31%)	1,233 (39%)	2,402 (45%)
	No Spouse/Partner	2,722 (25%)	183 (19%)	483 (30%)	619 (20%)	1,437 (27%)
	**% FRIENDS USING**					
	Almost None	2,110 (19%)	149 (16%)	618 (38%)	410 (13%)	933 (18%)
	25%	2,499 (23%)	256 (27%)	412 (25%)	687 (22%)	1,144 (22%)
	50%	2,716 (25%)	227 (24%)	230 (14%)	895 (29%)	1,364 (26%)
	75%	2,432 (22%)	170 (18%)	157 (10%)	817 (26%)	1,288 (24%)
	100%	826 (07%)	92 (10%)	69 (04%)	229 (07%)	436 (08%)
	No Friends	437 (04%)	61 (06%)	134 (08%)	89 (03%)	153 (03%)
	**% COWORKERS USING**					
	Almost None	2,046 (19%)	131 (14%)	387 (25%)	485 (16%)	1,043 (20%)
	25%	1,758 (16%)	161 (17%)	239 (15%)	551 (18%)	807 (15%)
	50%	1,386 (13%)	119 (13%)	144 (09%)	534 (17%)	589 (11%)
	75%	974 (09%)	87 (09%)	91 (06%)	389 (13%)	407 (08%)
	100%	300 (03%)	31 (03%)	21 (01%)	117 (04%)	131 (03%)
	No Coworkers	4,370 (40%)	407 (43%)	686 (44%)	1,018 (33%)	2,259 (43%)

*Tests for all Race by Sex interaction p < 0.001 based on Likelihood-ratio test*.

Compared with EA participants, AAs reported approximately one less year of regular smoking, despite being ~2 years older. AA men started smoking on average 1.2 years later, and started smoking regularly on average 1.4 years later relative to EA men; AA women started smoking 2.0 years later, and started smoking regularly 2.1 years later, relative to EA women. Compared with EA men, AA men reported smoking about 8 fewer cigarettes per day in the past year, their maximum daily number of cigarettes was 9 fewer, and that level was reached 0.6 years later. Compared with EA women, AA women reported smoking on average about 6 fewer cigarettes daily in the past year, their maximum daily number of cigarettes was about 13 fewer and that level was reached 1.7 years later. Consistent with these data, the average FTND total score was about 0.9 point lower in AA men, and about 0.7 point lower in AA women, compared with EA men and women, respectively. Compared with EAs, four times as many AA men and three times as many AA women smoked menthol cigarettes (Table 2).

### Tobacco Use by Others

All others' tobacco use measures were also significantly different by Race × Sex at *p* < 0.001. Approximately 6% more women than men, and 14% more EA than AA participants stated that their spouse/partner used tobacco. More men than women and a greater number of EAs than AAs reported that at least half of their co-workers used tobacco. Twenty-eight percent of AA women indicated that at least half of their friends used tobacco, compared with 52–62% for the other groups (Table 2).

### Multivariable Predictors of Tobacco Use Factors

After adjusting for key confounding variables in the multivariable regression analyses, the interactive effect of race and sex on the tobacco use outcomes remained significant for all measures except age starting regular smoking, number of years with regular smoking, and age when reached maximum daily rate (Table 3). Whether within men or women, AA and EA participants differed significantly on all tobacco use outcomes. Likewise, men and women differed in almost all outcomes within EA and AA subgroups, except that for AAs no sex difference existed for menthol use or FTND score. AA men and AA women were 3–4 times more likely to smoke mentholated cigarettes than their EA counterparts. AA men smoked regularly for an average of 2.5 years less than EA men; AA women smoked regularly for an average of 2.9 years less than EA women. Compared with EA men, AA men reported reaching their maximum daily number of cigarettes about 1.3 years older. Otherwise the adjusted values were similar to the unadjusted ones.

**Table 3:** Adjusted predictors of tobacco use for race by sex subgroups.

**Factor**	**Race comparisons** **within sex**		**Sex comparisons** **within race**		***p*-value for Race x Sex**
	**Men** **AA vs. EA**	**Women** **AA vs. EA**	**AA** **Men vs. Women**	**EA** **Men vs. Women**	
Menthol use	3.98 (3.61, 4.40)	3.10 (2.90, 3.32)	ns	0.80 (0.71, 0.89)	0.0040
FTND score	−0.99 (−1.20, −0.79)	−0.59 (−0.76, −0.42)	ns	0.20 (0.07, 0.33)	0.0022
Age 1^st^ cigarette	1.21 (0.78, 1.65)	1.76 (1.41, 2.11)	−1.46 (−1.94, −0.98)	−0.92 (−1.18, −0.65)	0.0479
Age started smoking regularly	1.53 (1.06, 2.00)	1.99 (1.61, 2.37)	−1.55 (−2.07, −1.03)	−1.09 (−1.38, −0.81)	0.1248 ns
Number of years regularly smoking	−2.46 (−3.17, −1.76)	−2.92 (−3.49, −2.35)	1.82 (1.04, 2.60)	1.37 (0.94, 1.80)	0.3096 ns
Average daily rate	−7.72 (−8.70, −6.75)	−5.51 (−6.29, −4.73)	1.49 (0.41, 2.57)	3.71 (3.13, 4.29)	0.0003
Maximum daily rate	−16.66 (−18.06, −15.25)	−11.93 (−13.06, −10.80)	4.11 (2.56, 5.66)	8.84 (7.99, 9.69)	< 0.0001
Age reached maximum daily rate	0.95^*^ (0.06, 1.84)	1.27 (0.54, 2.00)	−1.83 (−2.82, −0.83)	−1.51 (−2.05, −0.97)	0.5768 ns

All comparisons for race or sex comparisons p < 0.001 except

* p < 0.05;

*ns = non-significant (p > 0.05)*.

*For race comparisons, positive results indicate higher values for AAs; negative results indicate higher values for EAs*.

*For sex comparisons, positive results indicate higher values for men; negative results indicate higher values for women*.

*The effects of race and sex on tobacco use outcomes were estimated from multivariable logistic regression for menthol use, and multivariable linear regressions for other outcomes with Race x Sex interaction, adjusting for age, education, marital status, employment, insurance status, number of smokers in household, spouse/partner use of tobacco, and percent of friends and coworkers using tobacco. Results were expressed as odds ratios for binary menthol use, and mean differences for continuous outcomes along with their corresponding 95% CIs*.

With respect to sex-related effects, men reported earlier smoking initiation, as well as the age when smoking become regular as compared with women. Men also smoked a higher maximum daily number of cigarettes and reached this point earlier, as well-smoked a higher average daily number of cigarettes. Surprisingly, mean FTND score was lower for men, although the difference was small. EA men were 20% less likely than EA women to smoke mentholated cigarettes; no difference was observed between AA men and women (Table 3).

## Discussion

This study examined characteristics of a large group of participants enrolled in an intensive face-to-face tobacco dependence treatment program, with an emphasis on the influence of race and sex. An understanding of how patient subgroups differ may have important implications for further development and tailoring of treatment efforts to address the specific needs of our patients, and thus maximize the chances of successful outcome. This investigation supports previous work demonstrating high utilization of intensive specialist tobacco treatment options by individuals who present as highly nicotine dependent and complex (18, 24, 25).

An overview of the entire sample reveals a pattern of characteristics that depicts the average individual as middle-aged, high-school educated, married and poor or lower middle class. Participants were highly nicotine dependent, and had been smoking for over 25 years, thus representing a significant at-risk group for developing tobacco-related diseases. As expected, a fairly high percentage of participants endorsed at least one serious diagnosis known to be associated with chronic tobacco use. Most were either self- or family-/healthcare provider referred. In addition, a sizable portion indicated the presence of a mental health or substance dependence condition. Such a profile is indicative of individuals who are likely to have considerable difficulty quitting tobacco, particularly on their own, or when employing self-help or lower intensity interventions (26–29). These findings support the general premise that intensive treatment programs are needed to address the needs of this segment of the tobacco-using population, and available evidence indicates positive outcomes can be achieved [e.g., (25, 30)]. It is important to realize that that significant heterogeneity is present among those seeking treatment services. For example, a recent study in which characteristics of those enrolled in an internet-based smoking cessation trial were examined revealed these such individuals possessed a demographic profile that differed significantly from the population studied in the current study, particularly regarding indicators of socioeconomic status and basic demographics (e.g., age and race distributions) (31). Similarly, An et al. (24) reported increased age and levels of nicotine dependence among those receiving in-person treatment as compared with those in a web-based program.

Although this study did not evaluate treatment effects, differences across key characteristics suggest the value of developing tailored treatment efforts, based on an improved triaging algorithm. In this study, race effects were pronounced and have implications for determining the relative emphasis of specific treatment components. For example, more of our AA participants had limited education, which has implications for intervention presentation and resource development. All patients with lower reading levels can benefit from educational pamphlets at the appropriate reading level. Additionally, a good pamphlet is culturally sensitive. In addition, evidence suggests targeted information regarding NRT and other medications (32), and specific strategies on tailoring treatment programs (33) have been recommended. A greater focus on and consideration of menthol products makes sense, given the high rates of use among African-Americans, and its relationship to nicotine dependence (34–36). Differences in marital status distribution may warrant greater influence on dealing with home smoking policies, and conjoint treatment options. Of course financial status has many implications, including considerations of billing for services. The high rates of medical and psychiatric disorders point to many treatment issues, including medication choices, motivational considerations, and others. Observed sex differences also have implications for addressing the role of smoking among partners, a key factor is the likelihood of relapse (37, 38). Race differences in FTND score and average number of cigarettes per day highlight the need to tailor pharmacotherapy regimens; while this is typically considered on a case-by-case basis, subgroup-specific information can help programs prepare for necessary resources given the particular populations to be served. Differences in spouse, friend and coworker tobacco use can guide efforts related to optimal strategies for quitting and for relapse prevention, e.g., differential usage patterns within social contexts call for specific intervention strategies to deal with important triggers eliciting use. Possibilities include behavioral rehearsal strategies, conflict resolution options, as well as basic stimulus control and related approaches.

This study has several important strengths relative to previous work, including a very large N, a true community sample of tobacco users, the collection of numerous assessment measures, and the delivery of services at a variety of sites across Mississippi by Certified Tobacco Treatment Specialists (see www.ctttp.org). The primary limitations are that findings emerging from this Southeastern USA sample may be limited in terms of generalizability to other treatment populations and that a single, standardized treatment model was employed. This study did not address poly-tobacco product use, which may have an influence on some of the variables examined. In addition, while a common method for collecting information, the dependent measures were self-report, and thus subject to the usual considerations regarding such measures (e.g., recall bias, estimation errors, etc.).

In summary, the findings of this study verify that patients who enroll in an intensive tobacco treatment program present as generally complex, possessing a variety of characteristics which are generally associated with poorer outcome. A few sex-related differences were noted, and those that did emerge were expected. In contrast, a number of race and race x sex findings were observed, suggesting tailoring treatment options to these populations has the potential to improve outcomes. In general, careful evaluation of these and other characteristics should facilitate the development of program options likely to improve services for specific subgroups of patients in this generally high-risk population.

## Ethics Statement

Approved by the University of Mississippi Medical Center Institution Review Board. Direct consent was deemed unnecessary by waiver of consent approval.

## Author Contributions

TP was involved in the conception of the study, guide and interpret statistical analyses, supervised related clinical activity, and manuscript write-up. CS was involved in data collection, data analysis and interpretation, and manuscript write up. NG and MS assisted with data collection and manuscript development. HP assisted with data collection and manuscript write-up. OE, JM and AP performed data analysis. KC was involved in study design and manuscript write up.

### Conflict of Interest Statement

The authors declare that the research was conducted in the absence of any commercial or financial relationships that could be construed as a potential conflict of interest.
